# Antibody-mediated delivery of IL-10 inhibits the progression of established collagen-induced arthritis

**DOI:** 10.1186/ar2115

**Published:** 2007-01-29

**Authors:** Eveline Trachsel, Frank Bootz, Michela Silacci, Manuela Kaspar, Hartwig Kosmehl, Dario Neri

**Affiliations:** 1 Institute of Pharmaceutical Sciences, Department of Chemistry and Applied Biosciences, ETH Zurich, Wolfgang-Paulistrasse 10, CH-8093 Zurich, Switzerland; 2 Institute of Pathology, Helios Klinikum Erfurt, Nordhaeuser Strasse 74, D-99089 Erfurt, Germany

## Abstract

The antibody-mediated targeted delivery of cytokines to sites of disease is a promising avenue for cancer therapy, but it is largely unexplored for the treatment of chronic inflammatory conditions. Using both radioactive and fluorescent techniques, the human monoclonal antibodies L19 and G11 (specific to two markers of angiogenesis that are virtually undetectable in normal adult tissues) were found to selectively localize at arthritic sites in the murine collagen-induced model of rheumatoid arthritis following intravenous (i.v.) administration. The same animal model was used to study the therapeutic action of the L19 antibody fused to the cytokines IL-2, tumour necrosis factor (TNF) and IL-10. Whereas L19–IL-2 and L19–TNF treatment led to increased arthritic scores and paw swellings, the fusion protein L19–IL-10 displayed a therapeutic activity, which was superior to the activity of IL-10 fused to an antibody of irrelevant specificity in the mouse. The anti-inflammatory cytokine IL-10 has been investigated for the treatment of patients with rheumatoid arthritis, but clinical development plans have been discontinued because of a lack of efficacy. Because the antigen recognised by L19 is strongly expressed at sites of arthritis in humans and identical in both mice and humans, it suggests that the fusion protein L19–IL-10 might help overcome some of the clinical limitations of IL-10 and provide a therapeutic benefit to patients with chronic inflammatory disorders, including arthritis.

## Introduction

The therapeutic potential of recombinant cytokines is often limited by severe toxicities, even at low doses, thus preventing dose escalation and the establishment of a sufficient concentration at target tissues. In principle, monoclonal antibodies could be used to deliver cytokines to sites of disease, thus increasing their potency and sparing normal tissues. Indeed, several antibody–cytokine fusion proteins have been investigated for cancer therapy applications, often with impressive therapeutic results [1-4].

Components of the modified extracellular matrix (ECM) are particularly attractive for antibody-based targeting applications, because these antigens are often stable and abundant [5]. In the past, our group has focused its efforts on the development of human monoclonal antibodies, which recognise ECM components that are virtually absent in normal tissues but strongly expressed at sites of disease and have a prominent perivascular pattern of expression [6]. Splice isoforms of abundant ECM components seem to be particularly suited for antibody-mediated *in vivo *targeting applications. For example, the extra domai [7-10] and C domain of tenascin-C [11-13] are two of the most promising markers of angiogenesis, which can be targeted using the high-affinity monoclonal antibodies L19 and G11, respectively [14-19]. Several derivatives of the L19 antibody have exhibited impressive therapeutic activity in animal models of cancer [1,2,4,20-24] and angiogenesis-related ocular disorders [25]. In particular, the antibody–cytokine fusions L19–IL-2 and L19–tumour necrosis factor (TNF) and the radiolabelled antibody SIP(L19) (SIP, small immunoprotein) are currently in clinical development [26,27].

The antibody-mediated targeted delivery of cytokines is largely unexplored in arthritis and other chronic inflammatory diseases. Although it is well established that pro-inflammatory cytokines (such as TNF and IL-1) can have a negative effect on arthritis [28,29], anti-inflammatory cytokines might provide a therapeutic benefit. For example, IL-10 has been shown in a collagen-induced arthritis (CIA) mouse model to inhibit paw swelling and an increase in arthritic score [30-33]. Recombinant IL-10 was found to synergize with TNF-blocking antibodies [33] and has been tested in clinical trials in combination with methotrexate [34,35]. The clinical development of recombinant human IL-10 was discontinued because of a lack of efficacy of the compound in humans.

In this article, we show that both L19 and G11 antibodies display an impressive ability to selectively localize at sites of arthritis in the CIA mouse model. Furthermore, whereas L19–IL-2 and L19–TNF treatment led to increased arthritic scores and paw swelling compared with controls, the fusion protein L19–IL-10 displayed therapeutic activity, which was superior to the activity of IL-10 fused to an antibody of irrelevant specificity in the mouse. These findings might be of clinical significance because EDB has an identical sequence in humans and mice [7,36], is virtually absent in normal adult tissues [37] and is strongly expressed at arthritic sites in patients [38-40].

## Materials and methods

### Animal model

Male DBA/1 mice (8–12 weeks' old) were immunized by intradermal injection at the base of the tail with 200 μg of bovine type II collagen (MD Biosciences, Egg, Zurich, Switzerland) emulsified with equal volumes of Freund's complete adjuvant (MD Biosciences). The procedure was repeated 2 weeks after the first immunization, but incomplete Freund's adjuvant (MD Biosciences) was used to emulsify the collagen in the second procedure. Mice were inspected daily and each mouse that exhibited erythema and/or paw swelling in one or more limbs was assigned to an imaging or treatment study.

Arthritis was monitored using two disease indices (clinical score and paw swelling). For the clinical score, each limb was graded daily in a nonblinded fashion (0 = normal, 1 = swelling of one or more fingers of the same limb and 2 = swelling of the whole paw), with a maximum possible score of 8 per animal. Paw swelling was assessed every second day using a calliper to measure the thickness of each limb under isoflurane anaesthesia. The paw thickness of each animal was expressed as the mean value of all four paws.

### Immunohistochemical analysis

Frozen sections of arthritic paws were fixed in ice-cold acetone and stained for EDB. For immunohistochemical analysis, the L19 antibody was used in the miniantibody format (SIP(L19)). As secondary detection antibodies, we used rabbit antihuman immunoglobulin (Ig) E (Dako, Golstrup, Denmark) followed by biotinylated antirabbit IgG (Biospa, Milan, Italy) and streptavidin–alkaline phosphatase complex (Biospa). Fast red TRsalt (Sigma, Saint Louis, Missouri, USA) was used to develop the staining. Slides were counterstained with haematoxylin, mounted with Glycergel^® ^mounting medium (Dako) and analysed with a Zeiss Axiovert S100 TV microscope (Feldbach, Switzerland).

For immunofluorescence, a double staining for EDB and CD31 was performed. The following primary antibodies were used: SIP(L19) and rat antimouse CD31 (BD Pharmingen, Erembodegen, Belgium). As secondary detection antibodies, we used rabbit antihuman IgE (Dako), followed by Alexa Fluor 594 goat antirabbit IgG (Molecular Probes, Leiden, The Netherlands) for EDB and Alexa Fluor 488 goat antirat IgG (Molecular Probes) for CD31. Slides were mounted and analysed, as described above.

### Near-infrared imaging of arthritic paws

The selective accumulation of SIP(L19) and SIP(G11) in arthritic mice was tested by near-infrared imaging analysis, as described by Birchler and colleagues [41]. Briefly, purified SIP(L19), SIP(G11) and SIP(HyHEL10) were labelled using Alexa750 (Molecular Probes), according to the manufacturer's recommendations, and injected into the tail vein of arthritic mice. Mice were anaesthetized using ketamine, 80 mg/kg body weight, and medetomidine, 0.2 mg/kg body weight, and imaged in a near-infrared mouse imager 24 hours after injection [41].

### Biodistribution experiments

The *in vivo *targeting performance of SIP(L19) in arthritic mice was evaluated by biodistribution analysis, as described previously [14,17]. Briefly, purified SIP(L19), SIP(G11) and SIP(F16) were radio-iodinated and injected into the tail vein of arthritic mice (5 μg corresponding to 12 μCi SIP(L19), 5 μg corresponding to 10 μCi SIP(G11) and 5 μg corresponding to 11 μCi SIP(F16) per mouse). Mice were sacrificed 24 hours after injection and their paws were exposed to a phosphorimager screen (Fujifilm, Dielsdorf, Switzerland) for 1 hour and read in a PhosphorImager (Fujifilm BAS-5000).

### Cloning of L19–IL-10 and HyHEL10–IL-10

The human IL-10 gene was amplified from a commercial cDNA panel (Clontech, Basel, Switzerland) by PCR using the following primer sequences: a backward antisense primer, 5'-AGCCCAGGCCAGGGCA-CCCAGTCTG-3'; and a forward sense primer, 5'-GTTTCGTATCTTCATT-GTCATGTAGGCTTCTATG-TAGTTGATGAAGATG-3'. The resulting fragment was then further amplified using the following primer sequences: a backward antisense primer, 5'-TCGGGTAGTAGCTCTTCCGGCTCATCGTCCAGCGGCAGCCCAGGC-CAGGGCACC-3'; and a forward sense primer, 5'-TAATGGTGATGGTGATGGTGGTTTCGTATCTTCATT-GTCATGTAGGCTTC-3', which appended part of a 15 amino acid linker (SSSSG)_3 _at its N-terminal and a His_6 _tag, stop codon and NotI restriction site at its C-terminal.

The genes for the single-chain variable fragments (L19) and (HyHel10) were coamplified with a signal peptide using the following primer pairs, respectively: a backward antisense primer, 5'-CCCAAGCTTGTCGACCATGGGCTGGAGCC-3' and a forward sense primer, 5'-GAGCCGGAAGAGCTACTACCCGATGAGGAAGATTTGATTTCCACCTTGGTCCCTTG-3'; and a backward antisense primer, 5'-CCCAAGCTTGTCGACCATGGGCTGGAGCC-3' and a forward sense primer, 5'-GAGCCGGAA-GAGCTACTACCCGATGAGGAAGATTTTATTTCCAGCTTGGTCCCC-3'. Using this strategy, a *Hin*dIII restriction site was inserted at the N-terminal and a complementary part of the linker sequence was inserted at the C-terminal.

The single-chain Fv and IL-10 fragments were then assembled using PCR and cloned into the *Hin*dIII and NotI restriction sites of the mammalian cell-expression vector pcDNA3.1(+) (Invitrogen, Basel, Switzerland).

### Expression and purification of L19–IL-10 and HyHEL10–IL-10

HEK293 cells were stably transfected with the previously described plasmids and selection was carried out in the presence of G418 (0.5 g/l). Clones of G418-resistant cells were screened for expression of the fusion protein by ELISA using a recombinant EDB of human fibronectin or lysozyme as antigens and an anti-His_6_-tagged antibody (Sigma) for detection. The fusion proteins were purified from the cell-culture medium by affinity chromatography over antigen columns, as described previously [17,42]. The size of the fusion proteins was analysed in reducing and nonreducing conditions on SDS-PAGE and in native conditions by FPLC gel filtration on a Superdex S-200 exclusion column (Amersham Pharmacia Biotech, Dübendorf, Switzerland).

The *in vivo *targeting performance of L19–IL-10 in tumour-bearing mice was evaluated by biodistribution analysis, as described previously [14,17]. Briefly, purified L19–IL-10 was radio-iodinated and injected into the tail vein of female 129Sv mice grafted with a subcutaneous F9 tumour (3 μg corresponding to 4 μCi L19–IL-10 per mouse). Mice were sacrificed 24 hours after injection; the tumours and organs were weighed and counted.

Biological activity of human IL-10 was determined by its ability to induce the IL-4-dependent proliferation of MC/9 cells [43] using a colourimetric thiazole blue (MTT) dye-reduction assay (Sigma). In a 96-well microtitre plate, 10,000 MC/9 (murine mast cell line) (ATCC-LCG, Molesheim Cedex, France) cells/well in 200 μl of medium containing 5 pg (0.05 units)/ml of murine IL-4 (eBiosciences, San Diego, CA, USA) were treated for 48 hours with varying amounts of human IL-10. The human IL-10 standard and fusion proteins were used at a maximum concentration of 100 ng/ml IL-10 equivalents and serially diluted. To this, 10 μl of 5 mg/ml MTT was added and the cells were incubated for 3–5 hours. The cells were then centrifuged, lysed with dimethylsulfoxide (DMSO) and read for absorbance at 570 nm.

### Targeted delivery of cytokines to arthritic lesions

Immunocytokines L19–IL-2 and L19–TNF-α were prepared, as described previously [1,2]. Each mouse that exhibited erythema and/or swelling of one or more paws was randomly assigned to a treatment or control group and therapy was started. Mice were given an i.v. injection in the lateral tail vein with saline or L19–IL-2, L19–TNF-α or L19–IL-10 diluted in a volume of 200 μl of saline. The cumulative doses for the fusion proteins were 60 μg of L19–IL-2, 15 μg of L19–TNF and 450 μg of L19–IL-10 per mouse. The arthritic score was evaluated daily in a nonblinded fashion. A scoring system was used with the following scale: 0 = normal, 1 = swelling of single fingers or toes and 2 = pronounced oedematous swelling of the whole paw [29]. Swelling of the paws was measured every second day using a calliper and the paw thickness of each animal was expressed as the mean score of all four paws. The results are displayed as the mean ± standard error of the mean (SEM) for each group; each group contained seven mice. Experiments were performed in agreement with Swiss regulations and under a project license granted by the Veterinäramt des Kantons Zürich, Switzerland (180/2004).

### Comparison of targeted versus systemic application of IL-10

Each mouse that exhibited erythema and/or swelling of one or more paws was randomly assigned to a treatment or control group and therapy was started. Mice were given an i.v. injection in the lateral tail vein with saline, L19–IL-10 (3 × 150 μg) or HyHel-IL-10 (3 × 150 μg). Six mice were analysed per group. The arthritic score was evaluated daily in a nonblinded fashion and paw thickness was measured every second day. Experiments were performed in agreement with Swiss regulations and under a project license granted by the Veterinäramt des Kantons Zürich (180/2004).

### Statistical analysis

Data are expressed as the mean ± SEM. Differences in the arthritis score between different populations of mice were compared using a Student *t *test. *P *< 0.05 was considered significant.

## Results

### Histochemical analysis of arthritic paws

**Figure 1:**
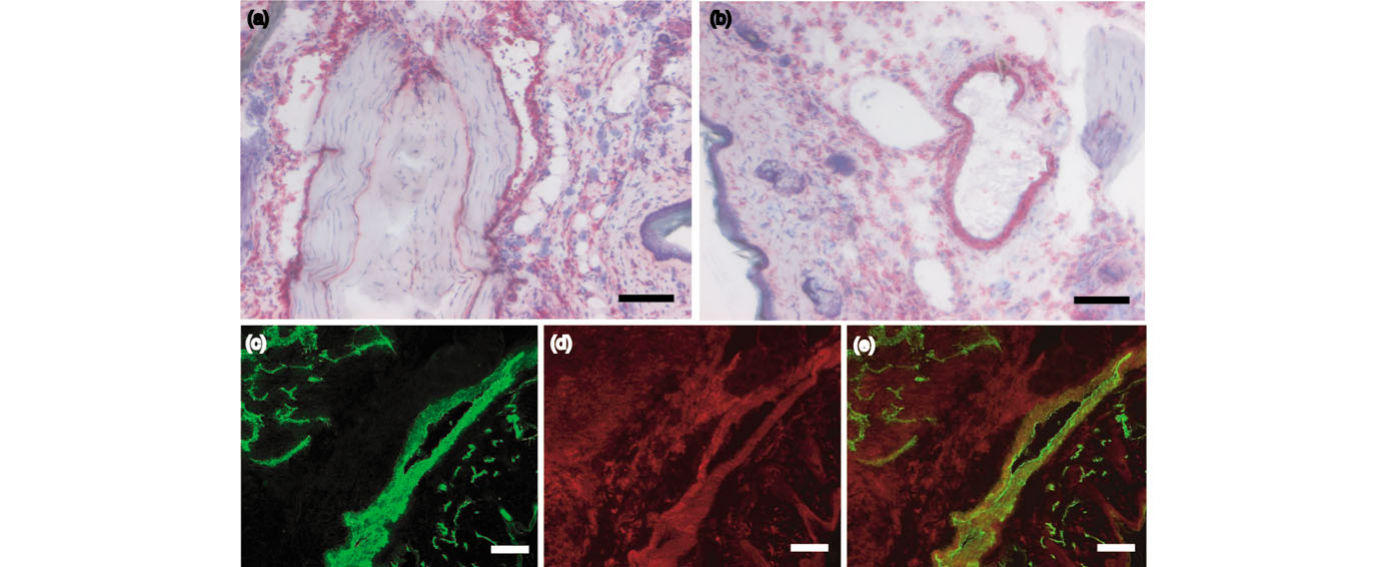
Histochemical analysis of arthritc paw sections. Immunohistochemistry with the small immunoprotein L19 on arthritic paw sections reveals expression of extra domain B of fibronectin (EDB) in the tunica synovialis and inflammatory infiltrates **(a) **and **(b)**. An immunofluorescent co-staining shows co-localisation of CD31 (green) and EDB (red) surrounding vascular structures **(c)–(e)**. Scale bars = 100 μm.

Expression of EDB-containing fibronectin was investigated by immunohistochemistry in inflamed paws from arthritic mice using the L19 antibody. EDB-containing fibronectin was detected in the tunica synovialis and stroma of inflammatory infiltrates (Figure 1a,b). Using immunofluorescence methods, the L19 antibody was furthermore shown to stain CD31-positive blood vessels (Figure 1c–e).

### The human monoclonal antibodies L19 and G11 selectively accumulate at sites of arthritis

We studied the *in vivo *targeting performance of L19 and G11 in miniantibody format (SIP) [14] in the CIA mouse model using both fluorescence and radioactivity for antibody detection. The SIP format consists of a single-chain Fv antibody fragment linked to the CH4 domain of human IgE, giving rise to a homodimeric protein of 80 kDa.

**Figure 2:**
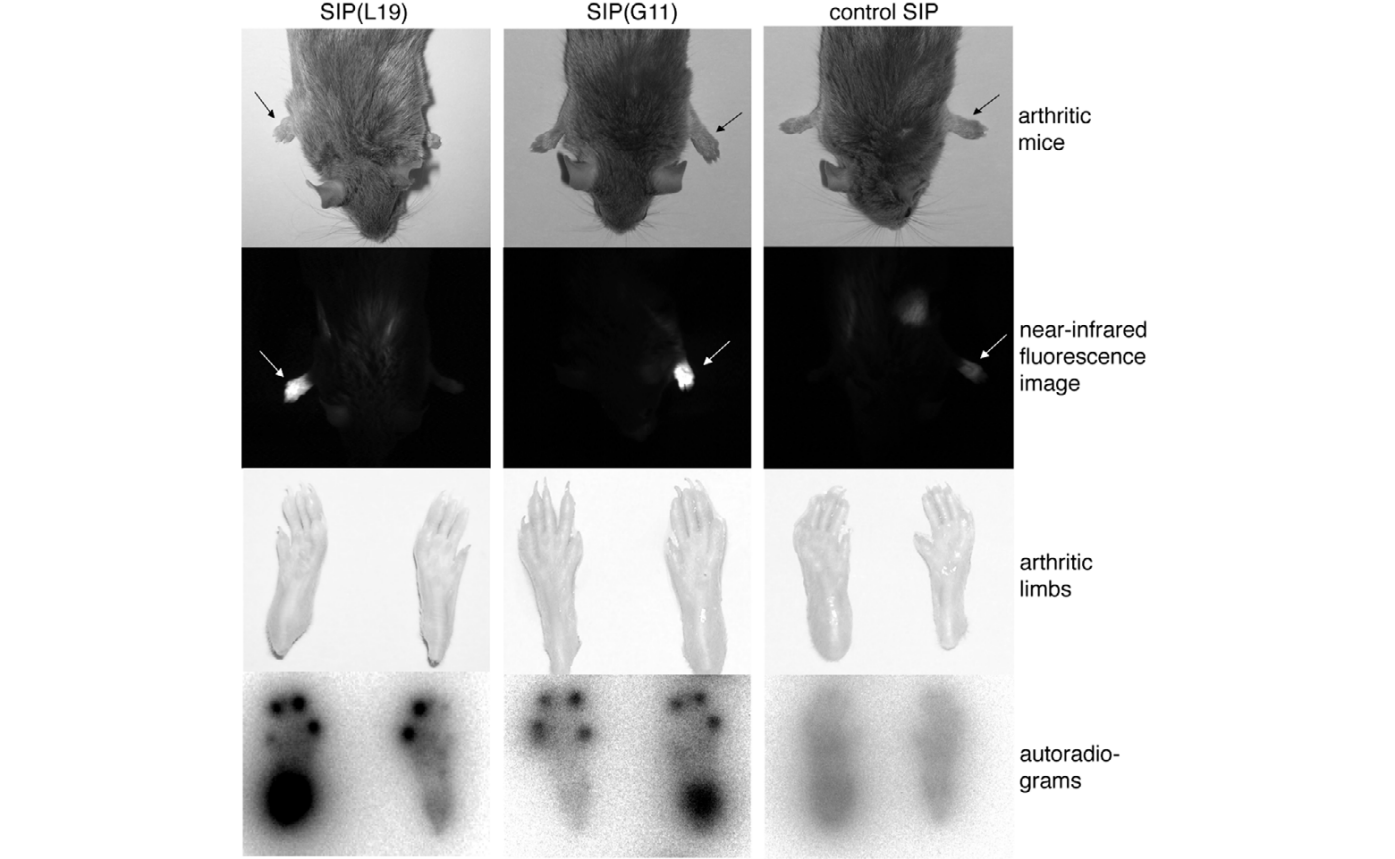
Investigation of the selective accumulation of the small immunoproteins L19 and G11 in inflamed limbs of arthritic mice. Arthritic mice were injected with SIP(L19)–Alexa750 (Molecular Probes, Leiden, The Netherlands), SIP(G11)–Alexa750 or the control antibody SIP(HyHEL10)–Alexa750. Near-infrared fluorescence imaging analysis was performed 24 hours after injection of the fluorescence-labelled antibodies. Arrows indicate grade 2 swelling in the front paws of the mice. In a similar experiment, arthritic mice were injected with ^125^I-labelled SIP(L19), SIP(G11) or SIP control. Uptake of radio-iodinated antibodies was analysed by phosphorimaging 24 hours after injection. The mouse injected with ^125^I-labelled SIP(L19) had an arthritic score of 2 in the left paw, whereas the right paw was classified as grade 1 arthritis. In the mouse injected with ^125^I-labelled SIP(G11) the left paw was classified as grade 1 arhritis, whereas the right paw was classified as grade 2 arthritis. In the mouse injected with ^125^I-labelled SIP(F16) the left paw was classified as grade 2 arthritis, whereas the right paw as grade 1 arthritis.

Arthritic mice were injected with SIP(L19), SIP(G11) or SIP(HyHEL10) labelled with the near-infrared dye Alexa750. Twenty-four hours after i.v. injection, animals were imaged using an infrared fluorescence imager [41], revealing a strong and selective antibody accumulation of SIP(L19) and SIP(G11) in the lesions present in the arthritic limb. By contrast, mice injected with SIP(HyHEL10), an antibody of irrelevant specificity in the mouse that was used as a negative control, displayed only a faint fluorescence signal, owing to nonspecific extravasation of the labelled antibody through the leaky vessels in the inflamed extremity (Figure 2).

Twenty-four hours after i.v. injection of SIP(L19) and SIP(G11), which were radiolabelled with iodine (^125^I), the mice were sacrificed and paws were imaged by autoradiography. A preferential accumulation of radioactivity was observed in the inflamed extremities of mice injected with SIP(L19) and SIP(G11), whereas no preferential antibody accumulation could be detected in mice exhibiting comparable grades of inflammation that had been injected with a SIP antibody of irrelevant specificity in the mouse. Biodistribution analysis performed with radiolabelled antibody preparations have previously shown that the percentage injected dose of antibodies in SIP format per gram of tissue typically lies below 1% 24 hours after injection, both for blood and for all major organs [14]. The ranges of lesion to nonaffected paw ratios measured by phosphorimaging were 3.3–8.5 for SIP(L19) and 2.0–4.9 for SIP(G11) (Figure 2).

### Cloning, expression and characterization of single-chain Fv–human IL-10 fusion proteins

**Figure 3:**
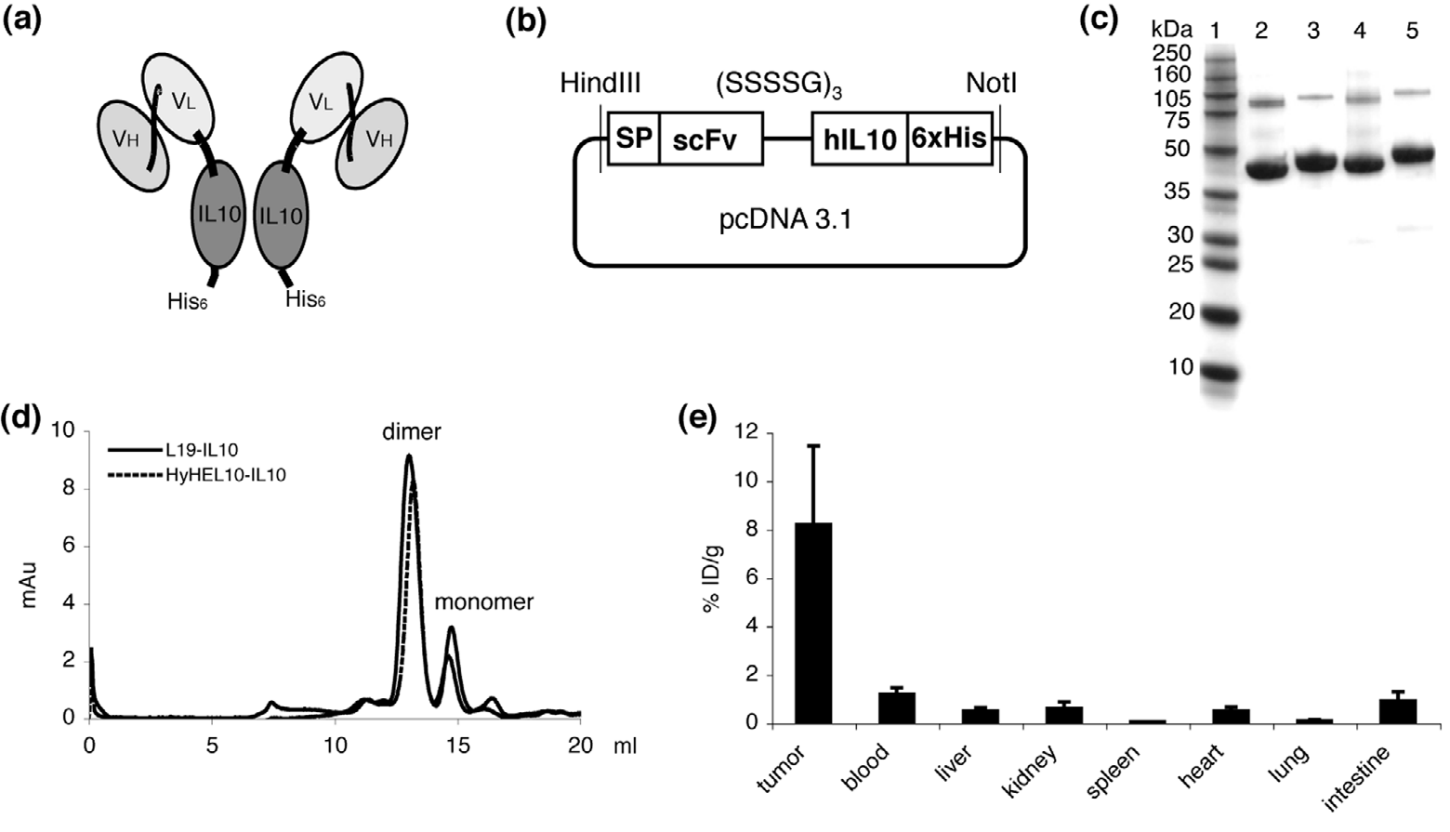
Cloning, expression and purification of L19–IL-10 and HyHel10–IL-10. **(a) **Schematic representation of single-chain variable fragment-IL-10 fusion proteins. **(b) **Schematic representation of a pcDNA3.1 vector (Invitrogen Basel, Switzerland) containing the essential elements of the L19–IL-10 or HyHEL10–IL-10 fusion proteins. The human IL-10 moiety was fused to the C-terminal of the single-chain Fv antibody fragment by the 15 amino acid linker (SSSSG)_3_. The secretion sequence at the N-terminal is required for secretion of recombinant proteins and the His_6 _tag at the C-terminal of human IL-10 was used for detection of the fusion proteins. **(c) **SDS-PAGE analysis of purified fusion proteins: lane 1, molecular-weight marker; lanes 2 and 3, L19–IL-10 under nonreducing and reducing conditions, respectively; lanes 4 and 5, HyHEL-IL-10 under nonreducing and reducing conditions, respectively. Monomeric fusion proteins are expected to have a molecular weight of 46 kDa. **(d) **The size-exclusion chromatography profile of purified L19–IL-10 (Superdex 200, GE Healthcare, Duebendorf, Switzerland). The peak eluting at a retention volume of 13 ml corresponds to the noncovalent homodimeric form of L19–IL-10, the smaller peak eluting at a retention volume of 16 ml corresponds to the monomeric fraction. (e) Biodistribution profile of L19–IL-10 in 129Sv mice grafted with a subcutaneous F9 tumour (*n *= 4). L19–IL-10 was labelled with ^125^I and administered by intravenous (i.v.) injection into tumour-bearing mice (3 μg corresponding to 4 μCi L19–IL-10 per mouse). Mice were sacrificed 24 hours after injection and the tumours and organs were weighed and counted. Values are displayed as percent injected dose per gram (%ID/g); standard errors of the means (SEMs) are indicated.

To investigate the effect of targeted delivery of cytokines to sites of inflammation, arthritic mice were injected with different L19–cytokine fusion proteins. The cloning and expression of the fusion proteins between single-chain Fv fragments of the L19 antibody (Fv(L19)) and IL-2 or TNF has been described before [1,2]. To test the therapeutic potential of a targeted version of the anti-inflammatory cytokine IL-10, we produced the fusion protein between single-chain Fv(L19) and IL-10 using a gene-fusion approach (L19–IL10; Figure 3a,b). In a similar manner, we produced a fusion protein between the negative-control antibody single-chain Fv(HyHEL10) [44] and IL-10, to assess the therapeutic performance of proteins with comparable clearance rates but different targeting selectivity.

The human antibody fragments single-chain Fv(L19) and single-chain Fv(HyHEL10), preceded by a signal sequence required for secretion of recombinant proteins, were genetically fused to human IL-10 with a 15 amino acid linker between the C-terminal of the single-chain Fv fragment and the N-terminal of human IL-10. A His_6 _tag was appended to the C-terminal of the fusion proteins and the resulting antibody derivatives were expressed in stably transfected HEK293 cells. L19–IL-10 and HyHEL10–IL-10 were purified from the culture medium by affinity chromatography on antigen columns at yields of 1–2 mg/l. Both fusion proteins migrated at the expected size of 43 kDa in reducing and nonreducing conditions (Figure 3c). The size-exclusion chromatography profile of the purified fusion proteins showed a main peak eluting at 13.5 ml, corresponding to the biologically active heterodimer, and a lower peak eluting at 16 ml, corresponding to the monomer (Figure 3d). The *in vivo *targeting properties of a radio-iodinated preparation of L19–IL-10 were evaluated in a biodistribution experiment [17] in 129SvEv mice carrying subcutaneous F9 teratocarcinomas [4]. Favourable tumor : organ ratios (ranging from 7 : 1 to 128 : 1) were observed 24 hours after i.v. administration (Figure 3e). The biological activity of human IL-10 was confirmed, by the ability of the two fusion proteins to induce IL-4-dependent proliferation of MC/9 cells [43], using the colourimetric MTT dye-reduction assay (data not shown).

### Effect of targeted delivery of cytokines to arthritic lesions

In a first-therapy experiment, we compared the potential of L19–IL-10 with L19–IL-2 and L19–TNF, using mice with CIA. Saline-injected mice were used as a control group. Mice received three injections every 48 hours, starting on day 1 after the onset of arthritis. The cumulative doses, which were equal to the doses previously used for tumour therapy experiments [1,2], were 60 μg of L19–IL-2 and 15 μg of L19–TNF. Each mouse received a dose of 450 μg of L19–IL-10 in this experiment and subsequent experiments with antibody–IL-10 fusion proteins, in line with IL-10 doses previously found to be active and nontoxic in mice [45].

**Figure 4:**
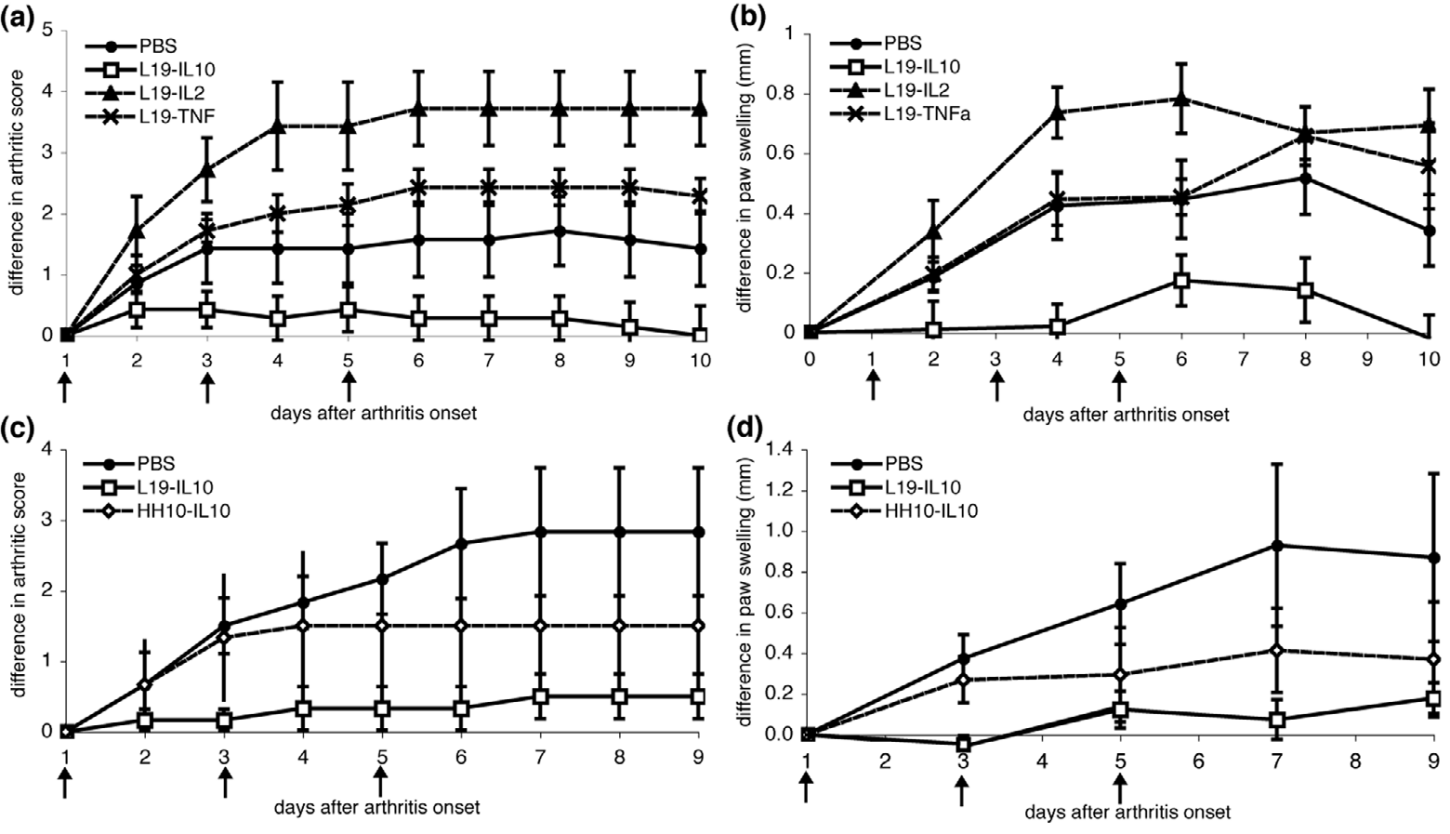
Selective delivery of cytokines to arthritic lesions. Arthritic mice were given an intravenous (i.v.) injection in the lateral tail vein with saline (black circles), L19–IL-2 (black triangles; dashed line), L19–tumour necrosis factor (TNF) α (crosses; dashed line) or L19–IL-10 (open squares) diluted in a volume of 200 μl of saline. Injections were started at day 1 after arthritis onset and then repeated every second day for three injections per animal, as indicated by the arrows. The cumulative doses for the fusion proteins were 60 μg of L19–IL-2, 15 μg of L19–TNF and 450 μg of L19–IL-10 per mouse. The arthritic score was evaluated daily and expressed as the mean ± the standard error of the mean (SEM) **(a)**. Paw swelling was measured every second day and paw thickness was expressed as the mean of all four paws of each animal. The results are displayed as the mean ± SEM for each group **(b)**. Each group contained seven mice. Comparison of targeted delivery and systemic application of IL-10 to arthritic mice. Arthritic mice were given an i.v. injection in the lateral tail vein with saline (black circles), L19–IL-10 (open squares) or HyHel10–IL-10 (crosses; dashed line) diluted in a volume of 200 μl of saline. Injections were started at day 1 of arthritis onset and then repeated every second day for three injections per animal, as indicated by the arrows. The cumulative doses for the fusion proteins were 450 μg per mouse. The arthritic score was evaluated daily and expressed as the mean ± SEM **(c)**. Paw swelling was measured every second day and paw thickness was expressed as the mean of all four paws for each animal. The results are displayed the mean ± SEM for each group **(d)**. Each group contained six mice.

L19–IL-10 had a clear therapeutic effect on both the arthritic score and paw swelling (*P *= 0.026; Figure 4a,b). The magnitude of this effect was similar to that observed with TNF-neutralizing antibodies in the same animal model [28,33]. By contrast, L19–IL-2 and L19–TNF led to a rapid and pronounced swelling of the affected limbs, which was more severe than the effect reported in the control (saline) group. None of the treated animals died or exhibited a weight loss of more than 15%, and arthritic parameters did not significantly worsen after the third antibody administration (Figure 4a).

### Comparison of targeted delivery compared with systemic application of IL-10

To demonstrate a therapeutic advantage of a targeted version of IL-10, compared with the untargeted cytokine, the two fusion proteins L19–IL-10 and HyHEL10–IL-10 were investigated in the CIA model. Similar to the previous experiment, arthritic mice were treated with three injections of L19–IL-10, HyHEL10–IL-10 or saline every second day, starting on the first day of arthritis onset. For both fusion proteins, the cumulative dose administered to each mouse was 450 μg. As expected, L19–IL-10 showed a significant therapeutic response compared with the control (saline) group (*P *= 0.037), with both the arthritic score and paw swelling remaining low until day 9 after arthritis onset (that is 4 days after the last injection). Consistent with previous observations of the therapeutic activity of IL-10 in this model, the nontargeted HyHEL10–IL-10 fusion protein displayed a therapeutic benefit compared with the saline control [33]; however, this was not as efficient as L19–IL-10 (Figure 4c,d).

## Discussion

The expression of fibronectin and tenascin-C isoforms at sites of arthritis opens new biomolecular avenues for the treatment of arthritic conditions. The L19 and G11 antibodies displayed the impressive ability to localize in affected limbs in a mouse model of CIA. By contrast, antibodies of irrelevant specificity in the mouse failed to accumulate (Figure 2). It is conceivable that the L19 and G11 antibodies could be used for the molecular imaging of arthritis in patients using positron emission tomography (PET) [46], near-infrared fluorescence [36,47], ultrasound [48] or magnetic resonance imaging (MRI) methodologies [49,50]. Fluorescence-based approaches might be particularly attractive for studying inflamed joints and planning immunophotodynamic therapy experiments using antibody-photosensitizer conjugates [21,25].

The ability of L19 and G11 to target arthritis *in vivo *raises the question of whether optimal antibody functionalization strategies could be used for therapeutic purposes. In the mouse model, the performance of L19–IL-10 was superior to untargeted IL-10 and similarly potent as TNF-neutralizing antibodies [28,33]. Previous reports of synergies between IL-10 and TNF-blocking agents and between IL-10 and methotrexate strongly suggest that L19–IL-10 should be clinically investigated (either alone or in combination) as a superior alternative to recombinant human IL-10. Clinical development of recombinant human IL-10 was discontinued because of a lack of efficacy in clinical trials, but there is a consensus that targeted delivery of this cytokine to sites of inflammation might lead to a dramatic enhancement of the therapeutic index [51,52].

The antibody-mediated targeted delivery of pro-inflammatory cytokines (such as IL-2, IL-12, TNF and interferon (IFN) γ) has resulted in impressive increase in the therapeutic index of these biopharmaceuticals, with striking curative activities in animal models of cancer [3,6,26,53]. In summary, the development of anti-inflammatory cytokine-antibody fusion proteins is a novel field of research, which promises to deliver novel biopharmaceutical agents of superior quality. Similar to cancer research, the identification and validation of pathology-associated targets is of fundamental importance to antibody development. Novel proteomic technologies derived from terminal perfusion of mouse models of pathology with biotinylation reagents, followed by a mass spectrometry-based comparative analysis, might be used in the future for the discovery of novel accessible markers of arthritis [54,55].

## Conclusion

The results obtained with the EDB of fibronectin and C domain of tenascin-C suggest that components of the modified ECM, generated by a mechanism of alternative splicing and displaying restricted patterns of expression in normal organs, might be ideal candidates for the development of antibody-based therapeutic strategies. These findings might be of clinical significance because the EDB has an identical sequence in humans and mice [7,36], is virtually absent in normal adult tissues [37] and is strongly expressed at arthritic sites in patients [38-40].

## Abbreviations

CIA = collagen-induced arthritis; ECM = extracellular matrix; EDB = extra domain B of fibronectin; ELISA = enzyme-linked immunosorbent assay; FPLC = fast performance liquid chromatography; IFN = interferon; Ig = immunoglobulin; IL = interleukin; i.v. = intravenous; MRI = magnetic resonance imaging; MTT = thiazole blue; PCR = polymerase chain reaction; scFv = single chain variable fragment; SEM = standard error of the mean; SIP = small immunoprotein; TNF = tumour necrosis factor.

## Competing interests

DN is a cofounder and shareholder of Philogen SpA, Siena, Italy; L19–IL-10 is patented and belongs to Philogen SpA. The remaining authors declare that they have no competing interests.

## Authors' contributions

ET participated in designing the study, cloned, produced and characterized the novel IL-10 fusion proteins, performed the animal experiments, contributed to the interpretation of the data and assisted in preparing the manuscript. FB set up the animal model in our laboratory and contributed essentially to the animal experiments. MS developed the G11 antibody and contributed to the *in vivo *targeting studies. MK participated in characterizing the novel IL-10 fusion proteins and assisted in the animal experiments. HK provided technical assistance for the histochemical analysis of the treated specimens. DN participated in conceiving the study, supervised the experiments, was involved in data interpretation and prepared the manuscript. All authors read and approved the final manuscript.
